# Open-label pilot study using hydroxytyrosol as dietary supplements in patients with mitochondrial diseases

**DOI:** 10.1186/s13023-025-03795-0

**Published:** 2025-06-06

**Authors:** Tsz Sum Wong, Emily Man, Ian Chi Kei Wong, Shirley Xue Li, Hui Zhi, Ka Man Carmen Yeung, Ka Yee Yip, Anna Ka Yee Kwong, Kiran Moti Belaramani, Suet Na Wong, Anne Mei Kwun Kwok, Miu Mak, Toby Chun Hei Chan, Pui Yee Lau, Yee Ling Elaine Kan, Kwok Chun Wong, Freddie Poon, Vansie Kwok, Chun Yin Dick Li, Paul Lau, Sze Man Lam, Vanessa Chu, Chi Fung Godfrey Chan, Cheuk Wing Fung

**Affiliations:** 1 Department of Paediatrics and Adolescent Medicine, Princess Margaret Hospital, Kowloon, Hong Kong; 2 Department of Paediatrics and Adolescent Medicine, The University of Hong Kong, Hong Kong SAR, China; 3 Department of Pharmacology & Pharmacy, The University of Hong Kong, Hong Kong SAR, China; 4 Biostatistics and Clinical Research Methodology Unit, School of Public Health, The University of Hong Kong, Hong Kong SAR, China; 5 Dietetics Unit, Allied Health Service, Hong Kong Children’s Hospital, Hong Kong SAR, China; 6 Department of Paediatrics and Adolescent Medicine, Hong Kong Children’s Hospital, Hong Kong SAR, China; 7 Chemical Pathology Laboratory, Department of Pathology, Hong Kong Children’s Hospital, Hong Kong SAR, China; 8 Department of Radiology, Hong Kong Children’s Hospital, Hong Kong SAR, China; 9 Pharmacy Department, Hong Kong Children’s Hospital, Hong Kong SAR, China; 10 Department of Anaesthesiology and Perioperative Medicine, Hong Kong Children’s Hospital, Hong Kong SAR, China; 11 Research Office, Hong Kong Children’s Hospital, Hong Kong SAR, China

**Keywords:** Mitochondrial diseases, Pilot study, Hydroxytyrosol, MELAS

## Abstract

**Background:**

Mitochondrial Diseases (MDs) refers to a heterogeneous group of inherited metabolic disorders resulting in defective cellular energy production due to abnormal oxidative phosphorylation (OXPHOS) caused by pathogenic mitochondrial DNA or nuclear DNA variants. As mitochondria are pivotal for cell bioenergetics, MDs could potentially affect multisystem, leaving a devastating and life-threatening impact. The treatment of MDs present significant challenges due to the complexity of the disease and the wide heterogeneity of its molecular defects. Thus, the need for innovative and more comprehensive therapeutic approaches is evident.

**Methods:**

This longitudinal, open-label study was a pilot trial involving 9 paediatric MD patients, aiming to gain a better understanding on the impact of hydroxytyrosol (HT) on the clinical outcomes of MD patients and to assess the feasibility and logistics of using HT as a dietary supplement for MD patients. Subjects received HT daily as dietary supplements for 12 months. Following this period, patients were then randomly assigned to either discontinue HT or continue receiving HT as their dietary supplements for an additional 6 months. Outcome measures that were assessed included the International Paediatric Mitochondrial Disease Scores, biochemical parameters, and quality of life assessments.

**Results:**

Among the outcome measures assessed, HT supplementation demonstrated the most considerable impact on improving the health-related quality of life according to the PedsQL scoring system and potential effects on a subgroup of MD patients with Mitochondrial encephalopathy, lactic acidosis, and stroke-like episode (MELAS).

**Discussion:**

This study demonstrated that HT supplementation resulted in improvement in health-related quality of life in MD patients, while the subgroup of MELAS patients showed additional potential beneficial effect from HT use. As a pilot trial, this study importantly highlighted HT’s tolerability in MD patients, which would facilitate trials of larger scale to be performed in the future.

**Conclusion:**

This study highlights the use of HT as a health supplement and its potential therapeutic effects in paediatric patients diagnosed with MDs, especially in MELAS patients. The results lay the foundation for future large-scale clinical trials. Consequently, further clinical intervention studies and investigations into HT’s potential therapeutic mechanisms at the molecular and intercellular levels are strongly encouraged.

**Supplementary Information:**

The online version contains supplementary material available at 10.1186/s13023-025-03795-0.

## Introduction

Inherited metabolic disorders (IMDs) are a heterogeneous group of disorders causing impairment of a biochemical pathway that have a defined or assumed primary genetic cause, regardless of whether the genetic material was inherited from a parent or appeared de novo [1]. Although any given IMD is considered a rare disease, as a group they may cause significant morbidity and even mortality. Mitochondrial diseases (MDs) are the commonest group of IMDs, with a minimal prevalence of 1 in 20,000 [2].

Mitochondrion, as the central organelle in cellular metabolism, houses a variety of metabolic reactions and performs a multitude of tasks, including combating the production of reactive oxygen species and generating energy as adenosine triphosphate (ATP) by means of the electron-transport chain and the oxidative phosphorylation (OXPHOS) system [3]. Any abnormal activities and discrete disruption of mitochondria are not only pivotal for cell bioenergetics, but also decide cell fate, including cellular proliferation, differentiation, autophagy and ultimately, cell death. Thus, it is not surprising that there is a direct or indirect involvement of mitochondria in the pathophysiology of primary MD [4].

MDs refer to a heterogeneous group of disorders resulting in defective cellular energy production due to abnormal OXPHOS system caused by pathogenic mitochondrial DNA (mtDNA) or nuclear DNA (nDNA) variants [5]. As mitochondria are pivotal for cell bioenergetics, MDs could potentially affect multiple body systems Systems affected include the brain, the heart, the muscles, and so on, leaving a devastating and life-threatening impact.

The treatment of MDs remains challenging despite ongoing therapeutic research. These challenges are largely due to the complexity and heterogeneity of MDs [2]. With the advent of next-generation sequencing, over 300 mitochondrial genes have been implicated in causing human diseases, and each of which presents a unique molecular defect. This wide heterogeneity of molecular defects further complicates and hinders the development of therapeutic strategies. For now, only a minority of MDs such as coenzyme Q10 biosynthesis disorders, disorders of riboflavin transport and metabolism, pyruvate dehydrogenase deficiency and SLC19A3 deficiency involving a thiamine transporter, are potentially treatable by oral supplements or dietary manipulations [6]. The majority of them are, however, without any cure. Thus, the need for fast and accurate strategies is crucial to identify novel treatment modalities for MDs. This urgency is due to the progressive nature of the disease, where any delay in treatment could potentially lead to the worsening and irreversibility of symptoms.

The majority of potential treatment strategies for MDs primarily focus on either enhancing mitochondrial function or managing the consequences of mitochondrial dysfunction. Certain therapies target the increase of respiratory chain substrate or the enhancement of electron transfer within the respiratory chain, while others attempt a biochemical bypass of specific respiratory chain complexes [3]. To find a cure for MDs, the need for innovative and more comprehensive therapeutic approaches is evident.

Extra-virgin olive oil (EVOO), rich in phenolic compounds, has been identified as a potential dietary supplement in protecting cells against oxidative damage and improving mitochondrial dysfunction [7]. Ongoing research suggests that daily consumption of EVOO, abundant in polyphenols, can enhance mitochondrial membrane fluidity and ATPase activity in patients with relapsing-remitting multiple sclerosis, a neurodegenerative disease associated with mitochondrial dysfunction [8]. EVOO has also been shown to improve oxidative stress biomarkers and motor coordination related to aging [9]. These benefits are attributed to phenolic compounds such as oleuropein, oleocanthal, hydroxytyrosol (HT), and tyrosol present in EVOO, which exhibit potent antioxidant properties against oxidative stress in brain tissues and protective effects on both acute and chronic neurodegenerative diseases [10]. These findings underscore the potential of phenolic compounds in EVOO as therapeutic agents in neurodegenerative diseases and set the stage for further exploration of their therapeutic potential. To elucidate the mechanisms by which these antioxidant phenolic compounds influence mitochondrial function, in vivo and in vitro experiments have been carried out. These experiments demonstrated the compounds’ protective effects on mitochondria by restoring mitochondrial enzymatic activities [11, 12], stimulating mitochondrial biogenesis and function, and improving complex activity and oxygen consumption [13]. Further in vitro studies on human endothelial cells also revealed that HT could ameliorate endothelial function under inflammatory conditions through the improvement of various aspects of mitochondrial function, including membrane potential, ATP synthesis, superoxide production, and mitochondrial biogenesis [14].

These findings have spurred interest in the potential role of phenolic compounds in the treatment of MDs. Preliminary and unpublished in vitro studies have been carried out in our research team using the skin fibroblasts for MD patients. Upon treating with HT, a dose-dependent reduction of reactive oxygen species production and an increase in cell viability upon exposure to hydrogen peroxide have been observed, warranting further investigation to HT as a potential therapeutic agent of MDs. Clinically, a patient with Mitochondrial encephalopathy, lactic acidosis, and stroke-like episode (MELAS) was self-prescribed on oleuropein. It was observed that the patient exhibited an event-free period from recurrent stroke-like episodes that directly correlated with the intake of oleuropein, as any discontinuation of the supplement led to a resurgence of the episodes, and resumption of the supplement consequently made the patient attack-free again.

The aforementioned findings, although promising, are based on preliminary observations from a small sample size and have not yet undergone a proper clinical trial. To substantiate these findings and better understand the impact of HT on the clinical outcomes of MD patients, it is essential to initiate a well-designed, proof-of-concept clinical trial. The present study aims to serve as a pilot investigation to assess the feasibility and logistics of using HT as a dietary supplement for MD patients. This trial will examine the effects of HT on clinical, biochemical, and radiological outcomes, as well as the patients’ quality of life under standardized conditions. Given the rarity of MDs, their high mortality rate, and varying degree of severity, patient recruitment may prove challenging. Nevertheless, the data gathered from this pilot study can serve as a valuable reference for future experimental designs, providing a foundation for establishing an effective dose of HT and requirements for larger clinical trials evaluating HT supplementation as a treatment for MDs.

## Materials and methods

This longitudinal, open-label study evaluated the response of paediatric patients with genetically confirmed MD to HT supplementation. All patients received a standardized dose of HT based on their age for the first part of the study, and were then randomized into 2 groups to either continue or discontinue the supplement. Outcome measures included internationally recognized disease scores, quality of life assessment, biochemical measures, radiological findings, and adverse effects. Data were collected at baseline and at multiple time points before and after randomization.

### Participants recruitment

This study, approved by the Research Ethics Committees under Hong Kong Hospital Authority Central Instiutional Review Board (application HKCH-REC-2020-027), focused on paediatric patients aged 3 to 18 with confirmed pathogenic variants in genes that are associated with MD in the nuclear or mitochondrial genome. Only patients that can comply with study requirements and have provided written informed consent were included. Children below the age of 3 have been excluded owing to prior research findings stated in the “Proposed Dosage of HT for Mitochondrial Diseases” section. Patients who were participating or had participated in any clinical trial involving HT or HT-associated phenols as dietary supplements within 2 months from the commencement of our study date, had a medical condition which could be exacerbated by HT or HT-associated phenols, olive oil allergies, pregnancy, and/or non-compliance were also excluded from the study. Withdrawal criteria included participant request, protocol violation or being passed away due to disease progression. Thus, in this pilot study, a total of 9 participants aged between 3 and 18 years were eligible and recruited from the Metabolic Medicine Outpatient Clinic in the Hong Kong Children’s Hospital (HKCH) for the study.

### Proposed dosage of Hydroxytyrosol for mitochondrial diseases

The use of dietary supplements containing HT has been investigated in several clinical trials to determine their safety and efficacy. According to the European Food Safety Authority in 2017, “No Observed Adverse Effect Level (NOAEL)” of HT was 50 miligram (mg) per kilogram (kg) per day. It was concluded that HT is safe, excluding children under 36 months [15]. Food and Drug Administration in the United States had no questions about the use of HT as a food ingredient with an estimated daily intake of 19.6 to 30.5 mg from 2 to 18 years old [16, 17]. Together with the information collected from the literature sources, it is indicated that within the daily uptake of HT at 45 mg in adults aged 25 to 42 years old, obtained from the *Process Engineering Department of Puleva Biotech SA*,* Granada* in *Spain*, HT was well tolerated with a decrease in red blood cell folate levels and ferritin [18]. No effect on the markers of cardiovascular diseases, blood lipids, inflammatory markers, liver function test, and renal function test including electrolytes were observed [18]. Another clinical trial involving paediatric patients aged between 4 and 16 years old with non-alcoholic fatty liver disease used a combination of 7.5 mg HT, provided by *elaVida™ (DSM*,* Heerlen*,* The Netherlands)* and 10 mg vitamin E [19]. The treatment resulted in a decrease in insulin resistance, triglyceride levels, oxidative stress parameters, and steatosis grade, with no significant adverse side effects reported.

**Table 1:** Dosage of Hydroxytyrosol used in this study for patients of different ages

Age of Subject (years)	Daily Dose of HT ^1^ (mg)
3–10	10
11–18	30

^1^ HT was obtained from *Hytolive*^®^*(Genosa*,* Madrid*,* Spain)*, in either liquid or powdered form, and administered in a single daily dose

Given the potential use of HT in treating MDs, the following dosages of HT were used for the study, based on evidence and data from previous clinical trials in humans including paediatric patients and the NOAEL (see Table 1).

### Experimental design for clinical trial of Hydroxytyrosol treatment

This was a longitudinal, open-label study. Subjects received HT daily as dietary supplements for 12 months. Following this period, patients were then randomly assigned in a 1:1 ratio using Microsoft Excel to either continue receiving HT (Group 1) as their dietary supplements for an additional 6 months or discontinue HT (Group 2).

Subjects visited HKCH at various time points after starting the dietary supplements for clinical follow-up, functional assessment (including questionnaires), blood sampling, and neuroimaging as primary and secondary outcome measures. More frequent follow-ups and monitoring were conducted after the initiation and withdrawal of supplements. Data on primary and secondary outcome measures (excluding radiological evaluation) were therefore collected at baseline, as well as at 2 weeks, 6 weeks, 3-, 6-, 9-, and 12-months post-trial commencement, and 2 weeks, 6 weeks, 3 and 6 months post-randomization.

An open-label trial was chosen for this study due to the current unavailability of a matching placebo with the same properties as HT (e.g. taste, smell, colour) to maintain blindness. Consequently, a withdrawal design was utilized despite its potential limitations, such as the ease of accessibility for patients to continue using HT independently after being assigned for withdrawal, which may influence the outcome.

### Primary outcome measures

#### International paediatric mitochondrial disease scores (IPMDS)

IPMDS, previously established by *Radboud Centre for Mitochondrial Medicine* to monitor symptoms and signs of disease progression in MD patients aged 1–18 years old, has been used as a standard primary outcome measurement [20]. IPMDS adapted the Newcastle Paediatric Mitochondrial Disease Scale (NPMDS) to a more detailed and clinically relevant scoring system that covers more symptoms indicated by patients and parents as “burdensome,” and includes a functional domain to quantify the change in motor abilities. This scale was suggested to be a robust tool for the follow-up of children with MDs and aimed for the purpose in clinical trials. IPMDS has been endorsed to be an outcome measure to assess childhood primary mitochondrial myopathy upon the consensus of a group of experts from established centres of excellence in the management and diagnosis of MD in an international workshop [21].

In brief, IPMDS consists of 61 items categorised into 3 domains – Domain 1, 2 and 3. Domain 1 has 23 items providing the detailed insights into subjective complaints and symptoms which should be assessed by interviewing parents or caregivers. Domain 2 consists of 25 items assessed by physical examination. Domain 3 has 13 items of functional assessment obtained by physical/motor function evaluation. The detailed development, testing, manual and final version were provided as supplementary materials with the paper published by Koene et al. [20]. IPMDS was conducted by a clinician who was experienced in the care of MD patients (C.W.F.).

### Secondary outcome measures

#### Biochemical parameters

Biochemical analysis assessing standard biomarkers for MDs, such as blood gas measurements, plasma lactate and amino acids were performed using standardised protocols in the Department of Pathology, HKCH. Blood gases were measured through electrochemical analysis, which can reveal metabolic acidosis as an indicator of MD [22]. Plasma lactate levels were assessed using an automated enzymatic method, where elevations could suggest the presence of mitochondrial dysfunction due to a shift in the mitochondrial redox state [23, 24]. Plasma amino acids levels were measured using liquid chromatography tandem mass spectrometry (LC-MS/MS). Increased plasma amino acid levels, including alanine, glycine, proline, and threonine, may indicate alterations in the redox state caused by respiratory chain dysfunction [23]. Creatine kinase levels were analyzed by a spectrophotometric method.

#### Paediatric quality of life (QOL) assessment

The measurement model used for QOL assessment was the Paediatric Quality of Life Inventory version 4.0 generic core scale (PedsQL 4.0 generic) for measuring health-related QOL in children and adolescents either in a healthy state or having acute or chronic health conditions. PedsQL is commonly used in paediatric populations and has been validated in various disease groups, including some conditions that share common characteristics with MDs, such as cerebral palsy and spinal muscular atrophy [25, 26]. PedsQL in this clinical trial is considered an appropriate tool for assessing QOL. PedsQL comprises 5 versions of the same questionnaire for various age ranges – (a) toddler version for 2- to 4-year-olds group; (b) young child version between 5- to 7-year-olds; (c) child version for 8- to 12-year-olds; (d) adolescent version for aged 13 to 18; and (e) young adult version for above 18 to 23. The Cantonese Chinese versions are readily available for the first 4 versions upon request. Each version has a parent version for proxy report and a child version for self-report (except for the toddler version). Each version consists of 23 items with 4 scales (physical, emotional, social, and school functioning) and 3 summary scores (total, physical health summary, psychosocial health summary score).

#### Study of tolerability

Tolerability was assessed by evaluating the number of intervention-related adverse events in patients taking HT as dietary supplements. Adverse events were not reported in the literature.

#### Radiological evaluation

Magnetic resonance imaging (MRI) of the brain with spectroscopy was used to evaluate patients’ responses before the trial as baseline, 12 months after receiving HT, and 6 months after withdrawal or continuation of HT following randomization. The Radboud Centre for Mitochondrial Medicine Paediatric MRI score (RCMM-PMRIS) was used as the outcome measure [27]. The RCMM-PMRIS focuses on the 6 most described abnormalities in neuroimaging and defines the extent of brain involvement. This can help evaluate MD severity, disease progression, and the radiological effects of treatment. DICOM datasets of the MRI brain of all subjects were reviewed and scored by 2 experienced paediatric radiologists (Y.L.E.K and K.C.W) independently on Siemens syngo.via workstation. Any discrepancy in score is resolved by consensus in simultaneous review.

## Results

A total of 9 participants (age range 3–17 years old, median age 11 years old, M:F = 3.5:1) were recruited (HT01-HT09). Details of the molecular diagnoses of our cohort are listed in Table 2. Due to the small sample size, clinical characteristics of the 2 groups randomized to continuing or discontinuing HT supplement after 12 months did not match, including the severity of MD. We define Group 1 (*n* = 4, patient number HT01 to HT04) as patients who were treated with HT for 18 months and Group 2 (*n* = 5, patient number HT05 to HT09) as those withdrawn from HT after 12 months of treatment.

**Table 2:** Molecular diagnoses, gender, and age of starting HT in the cohort

Subject	Gender	Age of starting HT (years)	Molecular findings
HT01	M	3.69	*MSTO1* p.Tyr109Serfs*18; p.Tyr478Cys *GJB2* homozygous p.Val37ile
HT02	F	5.71	NC_012920.1:m.10191T > C Heteroplasmic level: Blood 76%, buccal 81%, and urine 84%
HT03	F	17.18	mtDNA deletion of 4.9kbp
HT04	M	12.93	NC_012920.1: m.3243 A > G Heteroplasmic level: Blood 67% and urine 97%
HT05	M	11.86	*TAZ* (NM_000116.3): c.718G > C p.(Gly240Arg)
HT06	M	17.54	NC_012920.1: m.3243 A > G Heteroplasmic level not available
HT07	M	8.86	*NDUFAF5* homozygous c.836T > G p.(Met279Arg)
HT08	M	15.35	NC_012920.1: m.3243 A > G Heteroplasmic level not available
HT09	M	11.87	NC_012920.1: m.8993T > C Heteroplasmic level: Blood 96% and urine 97%

### Primary outcome measures

#### International paediatric mitochondrial disease scores measurements

**Fig. 1:**
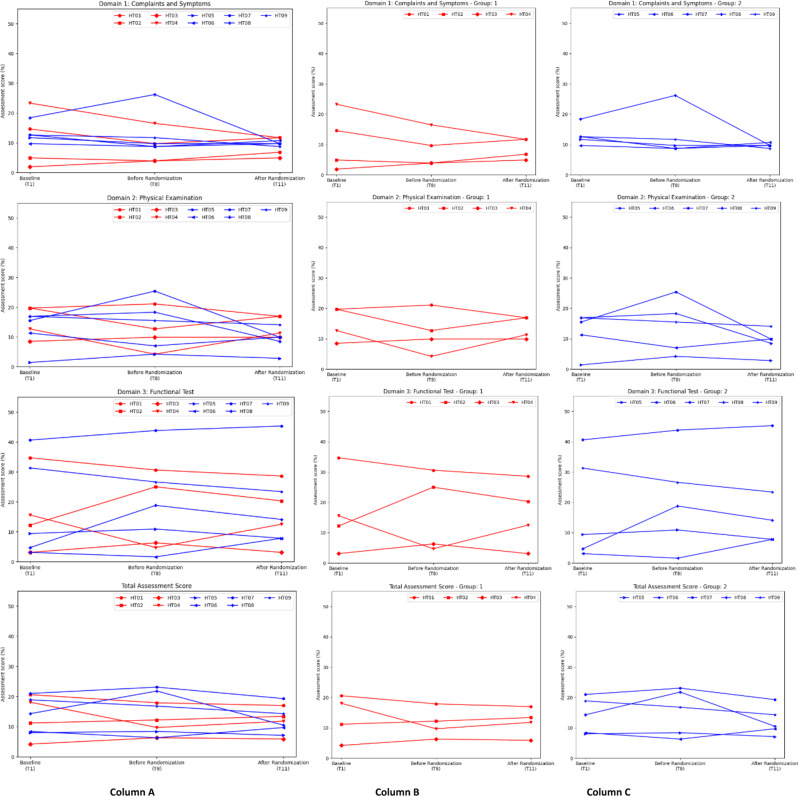
International Paediatric Mitochondrial Disease Scores (IPMDS) results. The figure presents the IPMDS, a standard primary outcome measurement comprising 61 items grouped into three domains: Domain 1 (Complaints and Symptoms), Domain 2 (Physical Examination), and Domain 3 (Functional Test), along with a total assessment score. Group 1 is in red while Group 2 is in blue. Column **A** shows the combined data from Group 1 and 2. Group 1 and 2 are then separately presented in Column **B** and **C** respectively

The IPMDS scores for Group 1 and 2 were examined at three different time points: baseline (T1), 12 months post-treatment with HT (T8), and 6 months post-randomisation (T11). A higher score indicates a worse clinical status. Upon examining various IPMDS domains of each patient at the 3 time points, most patients did not show a definite change in the IPMDS scores, with exceptions of the patients suffering from MELAS (Fig. 1). Also, no clear differences were observed between the 2 groups after HT was discontinued for Group 2 (See Fig. 1).

**Fig. 2:**
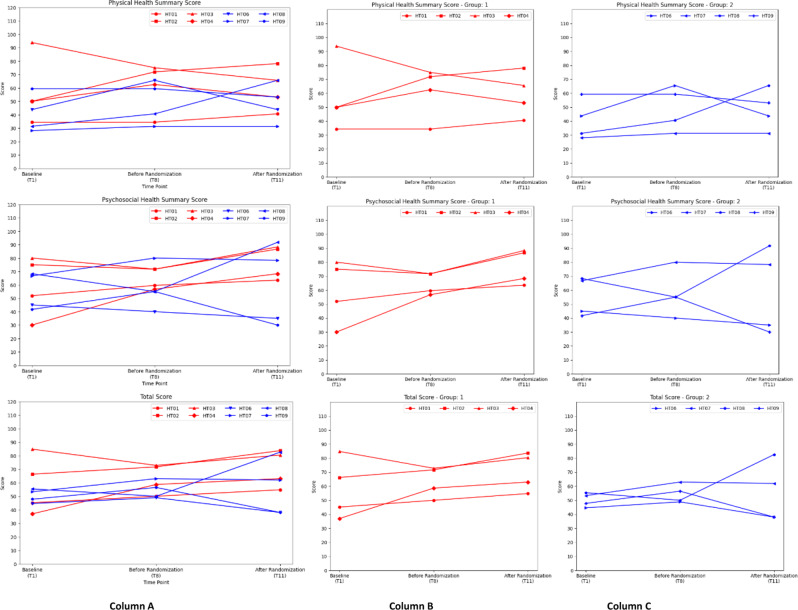
PedsQL (Paediatrics Quality of Life) Scores. The PedsQL results of Group 1 and 2 are categorized into physical, psychosocial and total scores. Group 1 is in red while Group 2 is in blue. Column **A** shows the combined data from Group 1 and 2. Group 1 and 2 are then separately presented in Column **B** and **C** respectively. Group 1 in general exhibited an increasing trend, indicating an improvement of health-related quality of life. In Group 2, 3 out of 4 patients (except HT08) showed an initial improvement after putting on HT but worsening after HT withdrawal

Patient HT04, who suffers from MELAS, showed an improvement in the total IPMDS score, from 18.1 to 9.7%, in the first 12 months of treatment. However, an additional six months of treatment did not yield further improvement, even though the final score of 11.8% remained lower than the baseline. His lactate level also showed a mild downtrend throughout the study (see section on Biochemical measures under Results).

Another patient with MELAS, HT08, showed initial worsening of IPMDS scores in the first 12 months of HT treatment in all domains (Domain 1: 18.4–26.2%; Domain 2: 16.9–18.3%; Domain 3: 4.7–18.8%; Total: 14.3–21.8%), followed by significant improvement in all domains after HT was stopped (Domain 1: 9.7%; Domain 2: 8.5%; Domain 3: 14.1%; Total: 10.5%). These improvements correlated with the time when ketogenic diet was commenced shortly after T8 due to recurrent status epilepticus without any evidence of stroke-like episodes. He remained seizure free afterwards with improved clinical parameters. Plasma lactate level also improved drastically to 1.9mmol/L (from 4.9mmol/L at T8) at the end of the study. This observation suggests that HT may not be effective in controlling epilepsy.

Patient HT06, who also suffers from MELAS, was the only patient from Group 2 that showed a deterioration in the IPMDS score after the withdrawal of HT, preceded by an initial improvement in the first 12 months of supplementation. At the 3 time points, his IPMDS scores improved across all domains in the first 12 months, followed by a mild worsening after HT withdrawal (Domain 1: 9.7–8.7 to 10.7%; Domain 2: 11.3–7 to 9.9%; Domain 3: 3.1–1.6 to 7.8%; Total: 8.4–6.3 to 9.7%). This also coorelates with an increase in cerebral white matter involvement after withdrawal of HT (see section on Radboud Centre for Mitochondrial Medicine Paediatric MRI Score under Results).

### Secondary outcome measures

#### Paediatrics quality of life score

The PedsQL scores, which indicate the health-related QOL in patients treated with HT, were analysed. PedsQL scores are weighted according to the generic core scales ranging from 0 to 100, with higher scores indicating better health-related QOL. Upon examination of the raw data (data not shown), patient HT05 in Group 2 was excluded from the study of PedsQL scores as marked discrepancies across various time points were noted, indicating that the questions could have been misinterpreted and answered inappropriately.

Overall, the administration of HT demosntrated a possible trend in improvng the health QOL despite the lack of definite clinical improvement indicated by the IPMDS scores throughout the study. Differences could be observed between the 2 groups after the withdrawal of HT in Group 2.

Throughout the 18-months period, patients HT01, HT02, and HT04 from Group 1 showed consistent improvement in the total score (Fig. 2). In fact, patient HT04 showed the greatest increase in total PedsQL scores among Group 1 patients, with 21.7 and 26.1 points increase from baseline at 12 months and 18 months of treatment, respectively. Among Group 1 patients, only HT03 had an overall decrease in total score after 18 months (4.3 points decrease) (Fig. 2).

In contrast, for patients HT06, HT07, and HT09 from Group 2, after showing initial improvement of the PedsQL total scores after 12 months of treatment, there was a decrease in the scores after HT was withdrawn (Fig. 2). The score changes from T1 to T8 and from T8 to T11 for these three patients are as follows; HT06: +4.3 points (T1 to T8) and − 10.9 points (T8 to T11), HT07: +9.8 points (T1 to T8) and − 1.1 points (T8 to T11), and HT09: +8.7 points (T1 to T8) and − 18.5 points (T8 to T11).

From Group 2, only patient HT08 showed an increase in total PedsQL score after 18 months (Fig. 2). However, this appeared to correlate with the initiation of ketogenic diet and the resultant improvement in seizure control, as his total PedsQL scores decreased by 5.4 points in the first 12 months, but improved drastically by 32.6 points between T8 and T11 (Fig. 2).

#### Radboud centre for mitochondrial medicine paediatric MRI score

The RCMM-PMRIS scoring system ranges between 0 and 3 per item. A higher score indicates more severe neuroimaging involvement (Table 3).

**Table 3:** Total MRI score using RCMM-PMRIS

Patient ID	Baseline (T1)	Before Randomization (T8)	After Randomization (T11)
**GROUP 1**			
HT01	2	2	1
HT02	5	5	5
HT03	6	6	6
HT04	6	7	7
**GROUP 2**			
HT05	0	0	0
HT06	3	4	7
HT07	3	3	3
HT08	12	13	13
HT09	5	5	5

Throughout the study, 5 of the patients (HT02, 03, 05, 07, 09) demonstrated static and stable cerebral involvement across the three timepoints (T1, T8 and T11), while no definite trend could be observed in the other 3 patients (HT01, 04, and 08). The only patient that had clear changes in the RCMM-PMRIS score was patient HT06 with MELAS, who showed an increase in cerebral white matter involvement after withdrawal of HT (raw data), where the score increased from 4 to 7 points.

#### Biochemical measures

The biochemical analysis aimed to assess the effectiveness of HT in enhancing mitochondrial activity and function by measuring biomarkers, including lactate, creatine kinase, acid-base status, and plasma amino acids in blood samples throughout the study period. Overall, HT supplementation did not result in definite beneficial or deterimental effects on the biochemical markers measured (Supplementary Table 1).

The only notable changes in plasma lactate levels were observed in the 2 MELAS patients, HT04 and HT08. As shown in Supplementary Table 1, patient HT04’s plasma lactate level dropped from 4.0mmol/L to 3.2mmol/L in the first 12 months, followed by a further drop to 2.5mmol/L after the additional 6 months of HT treatment. However, there was no corresponding improvement in the plasma alanine, glycine, proline, and threonine levels. So the downtrend of plasma lactate level needs to be interpreted with caution.

Patient HT08’s plasma lactate level remained high during the first 12 months of treatment (4.8mmol/L at T1 and 4.9mmol/L at T8), but dropped to 1.9mmol/L at the end of study. This correlated with the improvement of clinical status and seizure control after ketogenic diet initiation.

#### Tolerance study of Hydroxytyrosol

With regard to the tolerance to HT, it was well tolerated by all patients throughout the study period. No adverse effects were reported by the patients, suggesting a paramount implication for the possibility of extension of HT-related study in the future. The lack of adverse effects was consistent with findings of previous toxicological evaluation of HT use, which found no adverse effects even at higher doses of HT use [28].

## Discussion

This pilot study investigates the potential therapeutic effects of HT, a prevalent phenolic compound found in EVOO, on paediatric MDs. Although no previous clinical trials have been reported on the use of HT as a supplement or medical treatment in MDs, initiating a pilot trial could significantly enhance our understanding and evaluation of HT’s potential as a therapeutic strategy for MDs. Furthermore, the findings of this report may serve as a foundational reference for future large-scale experimental designs.

Among the various outcome measures assessed, HT supplementation demonstrated the most considerable impact on improving the health-related QOL, as evidenced by the higher scores in the PedsQL scoring system for most Group 1 patients throughout the 18 months of HT supplementation, while most Group 2 patients exhibited initial improvement during HT supplementation but worsening of the score after HT was withdrawn. The molecular mechanism responsible for these improvements remains unclear; however, it is hypothesized that HT’s involvement in exerting antidepressant-like effects may be attributed to its ability to reduce oxidative stress, suppress neuroinflammation, and enhance neurotrophic factor functionality [29]. This, in turn, could potentially lead to the observed improvements in psychosocial health status. However, to verify the accuracy of this hypothesis, additional studies are required to provide further insight into the underlying mechanisms of HT’s therapeutic effects. Alternatively, the observed improvement could also be attributed to a placebo effect, whereas the act of participating in a trial can make patients closely monitored with the hope of having a cure to MDs, which further boosted their morale and positively impact their overall well-being.

Even though there is no definite trend in other outcome measures between Group 1 and 2, subgroup analysis revealed potential effects of HT in 2 MELAS patients in our cohort (HT04 and HT06). Patient HT04 showed the most clear improvement, as evidenced by increase in his IPMDS and PedSQL scores, and reduction in plasma lactate level. As mentioned, his total IPMDS score improved in the first 12 months of treatment, but continuation for another 6 months caused mild worsening even though the score at the end of the study remained lower than that from the baseline. MRI findings remained stable. His plasma lactate level also revealed a downtrend from 4.0mmol/L to 2.5mmol/L, but should be interpreted with caution as the plasma alanine level did not show corresponding improvement. For patient HT06, he exhibited a mild improvement in all domains in IPMDS score during HT supplementation. Of note, his IPMDS scores worsened in all domains after treatment withdrawal, and was accompanied by an increase in cerebral white matter involvement.

On the other hand, patient HT08 showed initial worsening of clinical status in all IPMDS domains in the first 12 months of treatment. He had recurrent episodes of breakthrough status epilepticus but no strong evidence of stroke-like episodes, followed by improvement even after withdrawal of HT, which had likely resulted from dramatic improvement in seizure control after being put on ketogenic diet. This may show that HT does not have major effect on seizure control. The remote possibility of HT aggravating seizures in patient HT08 cannot be totally excluded, but this has not been reported in the literature and other patients with epilepsy in the cohort did not experience breakthrough seizures during HT treatment. Moreover, this patient had a history of intractable seizures even before this study. Another unpublished patient with MELAS, described in the “Introduction” section, remained free of stroke-like episodes while on oleuropein.

The observation of improvements seen in patient HT04, HT06, and the unpublished MELAS patient may indicate potential therapeutic effect of HT in this subgroup of MD patients. Such effect may have been masked by the intractable epilepsy in HT08. Larger scale and placebo-controlled trials are of great importance to ascertain the effect of HT on MELAS patients.

Prior studies have suggested the association of HT with antioxidant activity in neurodegenerative diseases, indicating its potential role in neuroprotection. The findings in patients HT04 and HT06 may indicate improved functional and structural connectivity between various brain regions, which this may be related to the association of HT with antioxidant activity in neurodegenerative diseases, indicating its potential role in neuroprotection [16]. In addition, HT’s ability to decrease the percentage of glycated hemoglobin and diastolic blood pressure, may exert a positive impact in reducing stroke [30].

Based on previous studies, it is believed that the high antioxidant efficiency of HT has been initially attributed to the presence of the o-dihydroxyphenyl moiety, which primarily acts as a chain breaker by donating a hydrogen atom to peroxyl-radicals (ROO*), introducing an intramolecular hydrogen bond in the phenoxy radical. Interestingly, an alternative mechanism might co-exist, providing additional antioxidant protection by enhancing the endogenous defense systems against oxidative stress through the activation of various cellular signalling pathways. One proposed mechanism involves the HT-mediated induction of phase II detoxifying enzymes via nuclear factor E2-related factor 2 (Nrf2) activation in different tissues. Furthermore, the interaction of HT with microRNAs (miRNAs) could potentially serve as a molecular target for eliciting its biological effects [17]. Therefore, further experimentation and investigation are recommended to elucidate the exact molecular mechanisms underlying these actions and to demonstrate how such characteristics may be beneficial in the therapeutic applications in MDs, especially in MELAS.

Considering the above findings and the on-going research studies related to HT’s antioxidant properties in in vitro trials, further research may be worthwhile using different biochemical approaches, such as enzymatic assays and western blotting, to examine the activity of major enzymatic components and their involvement in HT metabolism or action in antioxidant activity for therapeutic applications.

Overall, this report underscores the need for continued research into the potential benefits of HT supplementation in the context of paediatric MD treatment and contributes to the growing body of knowledge surrounding the therapeutic potential of HT in various health contexts. According to this and prior studies, HT could have potential therapeutic implications in MDs, especially MELAS. HT has been well tolerated without any adverse effects. Extension of this clinical trial in the future may involve a larger cohort of MELAS patients to be put on an even higher dose of HT for a longer period of time.

Despite the potential findings generated by this study, certain limitations must be considered. Primarily, the small sample size may limit the significance of the results. MDs are classified as rare diseases, with a global occurrence rate of approximately 1 in 20,000. Consequently, the number of diagnosed paediatric patients available for recruitment into the study is low. Statistical analysis is also limited by the small sample size of this study. Future research with larger and more homogeneous samples could provide further insights into the potential benefits of HT supplementation in MD patients.

The findings from this report imply HT supplementation may have a beneficial impact on health-related QOL in paediatric patients with MD, as evidenced by higher PedsQL scores. Additionally, based on clinical scores and MRI findings, subgroup analysis indicates that HT could offer specific advantages for patients with MELAS, likely due to HT’s high antioxidant efficiency and neuroprotective potential. Nevertheless, the need for ongoing research into HT’s benefits for paediatric MDs, contributing to the broader understanding of its therapeutic potential across various health contexts, is encouraged.

## Conclusions

This study highlights the use of hydroxytyrosol (HT), a major phenolic component in extra-virgin olive oil (EVOO), as a health supplement and its potential therapeutic effects in paediatric patients diagnosed with mitochondrial diseases (MDs). The results offer valuable insights and lay a solid foundation for future large-scale clinical trials. Future trials focusing on MELAS patient can be considered, given the beneficial effect seen in this subgroup of MD patients in our study. Futher clinical intervention studies and investigations into HT’s potential therapeutic mechanisms at the molecular and intercellular levels are strongly encouraged.

## Electronic supplementary material

Below is the link to the electronic supplementary material.

Supplementary Material 1

## Data Availability

All available data generated during this study are included in the published article.
