# Supplementary material for: Open-label pilot study using hydroxytyrosol as dietary supplements in patients with mitochondrial diseases
Source: Orphanet J Rare Dis. 2025 Jun 6;20:283. doi: 10.1186/s13023-025-03795-0 (PMC12142884; doi:10.1186/s13023-025-03795-0)
Supplement: Supplementary file 1 — Supplementary Material 1 [file 13023_2025_3795_MOESM1_ESM.docx]

| **Patient number** | **Biochemical measures in blood** | **Baseline (T1)** | **Before Randomization (T8)** | **After Randomization (T11)** |
| --- | --- | --- | --- | --- |
| **GROUP 1** | | | | |
| **HT01** | Lactate (mmol/L) | 1.2 | 0.9 | 0.8 |
|  | pH | 7.44 | 7.38^b^ | 7.39 |
|  | Serum HCO_3_ (mmol/L) | 22.7 | 26.1^b^ | 23.4 |
|  | CK (U/L) | 1240 | 1045 | 1169 |
|  | Plasma alanine (umol/L) | 235 | 189 | 201 |
|  | Plasma glycine (umol/L) | 156 | 167 | 174 |
|  | Plasma proline (umol/L) | 159 | 97 | 99 |
|  | Plasma threonine (umol/L) | 121 | 63 | 93 |
| **HT02** | Lactate (mmol/L) | 1.2 | 1.5 | 2.4 |
|  | pH | 7.35 | 7.38 | 7.4 |
|  | Serum HCO_3_ (mmol/L) | 22.9 | 24.5 | 25.5 |
|  | CK (U/L) | 77 | 134 | 180 |
|  | Plasma alanine (umol/L) | 351 | 351 | 474 |
|  | Plasma glycine (umol/L) | 165 | 155 | 181 |
|  | Plasma proline (umol/L) | 127 | 108 | 130 |
|  | Plasma threonine (umol/L) | 114 | 66 | 74 |
| **HT03** | Lactate (mmol/L) | 1.8 | 2.2 | 1.7 |
|  | pH | 7.32 | 7.36 | 7.34 |
|  | Serum HCO_3_ (mmol/L) | 20.5 | 21.8 | 23.8 |
|  | CK (U/L) | 108^a^ | 84 | 80 |
|  | Plasma alanine (umol/L) | 481 | 650 | 489 |
|  | Plasma glycine (umol/L) | 188 | 250 | 206 |
|  | Plasma proline (umol/L) | 141 | 195 | 161 |
|  | Plasma threonine (umol/L) | 131 | 209 | 153 |
| **HT04** | Lactate (mmol/L) | 4.0 | 3.2 | 2.5 |
|  | pH | 7.43 | 7.36 | 7.33 |
|  | Serum HCO_3_ (mmol/L) | 23.4 | 22.1 | 21.4 |
|  | CK (U/L) | NA | 139^b^ | 153 |
|  | Plasma alanine (umol/L) | 521 | 539 | 589 |
|  | Plasma glycine (umol/L) | 126 | 126 | 166 |
|  | Plasma proline (umol/L) | 309 | 253 | 276 |
|  | Plasma threonine (umol/L) | 62 | 76 | 77 |
| **GROUP 2** | | | | |
| **HT05** | Lactate (mmol/L) | 0.6 | 0.8 ^b^ | 0.8 |
|  | pH | 7.34 | 7.34 ^b^ | 7.35 |
|  | Serum HCO_3_ (mmol/L) | 26.2 | 27.7 ^b^ | 26.1 |
|  | CK (U/L) | 100 | 91 ^b^ | 93 |
|  | Plasma alanine (umol/L) | 311 | 383 ^b^ | 354 |
|  | Plasma glycine (umol/L) | 177 | 216 ^b^ | 193 |
|  | Plasma proline (umol/L) | 134 | 205 ^b^ | 164 |
|  | Plasma threonine (umol/L) | 139 | 203 ^b^ | 164 |
| **HT06** | Lactate (mmol/L) | 2.3 | 2.3 | 2.5 |
|  | pH | 7.37 | 7.41 | 7.35 |
|  | Serum HCO_3_ (mmol/L) | 28.4 | 24.2 | 26.3 |
|  | CK (U/L) | 123 | 138 | 123^c^ |
|  | Plasma alanine (umol/L) | 473 | 448 | 469 |
|  | Plasma glycine (umol/L) | 223 | 184 | 208 |
|  | Plasma proline (umol/L) | 202 | 167 | 123 |
|  | Plasma threonine (umol/L) | 105 | 110 | 94 |
| **HT07** | Lactate (mmol/L) | 2.9 | 2.3 | 1.7 |
|  | pH | 7.27 | 7.3 | 7.36 |
|  | Serum HCO_3_ (mmol/L) | 23.5 | 24.8 | 23.2 |
|  | CK (U/L) | 351 | 276 | 324 |
|  | Plasma alanine (umol/L) | 283 | 285 | 257 |
|  | Plasma glycine (umol/L) | 160 | 154 | 198 |
|  | Plasma proline (umol/L) | 133 | 147 | 125 |
|  | Plasma threonine (umol/L) | 83 | 84 | 110 |
| **HT08** | Lactate (mmol/L) | 4.8 | 4.9 | 1.9 |
|  | pH | 7.38 | 7.43 | 7.46 |
|  | Serum HCO_3_ (mmol/L) | 28 | 27.4 | 23.5 |
|  | CK (U/L) | 289 | 222 ^b^ | 247 |
|  | Plasma alanine (umol/L) | 676 | 587 | 329 |
|  | Plasma glycine (umol/L) | 161 | 103 | 145 |
|  | Plasma proline (umol/L) | 219 | 196 | 122 |
|  | Plasma threonine (umol/L) | 98 | 60 | 77 |
| **HT09** | Lactate (mmol/L) | 0.8 | 1.4 | 1.4 |
|  | pH | 7.34 | 7.37 | 7.34^c^ |
|  | Serum HCO_3_ (mmol/L) | 24.0 | 22.1 | 22.7^c^ |
|  | CK (U/L) | 77 | 162 | 115 |
|  | Plasma alanine (umol/L) | 423 | 365 | 512 |
|  | Plasma glycine (umol/L) | 190 | 204 | 216 |
|  | Plasma proline (umol/L) | 142 | 126 | 201 |
|  | Plasma threonine (umol/L) | 75 | 77 | 120 |

**Supplementary table 1: Biochemical measures.**

Abbreviations: *CK* Creatine Kinase, *HCO_3_* Bicarbonate, *NA* Not available, *T1* Time point 1, *T8* Time point 8, *T11* Time point 11.

^a^ Data from time point 2 (2 weeks from start of study), if available, was used to replace missing data from T1.

^b^ Data from time point 7 (12 months post-trial commencement), if available, was used to replace missing data from T8.

^c^ Data from time point 10 (3 months post-randomization), if available, was used to replace missing data from T11
